# Novel Variants in Individuals with *RYR1*-Related Congenital Myopathies: Genetic, Laboratory, and Clinical Findings

**DOI:** 10.3389/fneur.2018.00118

**Published:** 2018-03-05

**Authors:** Joshua J. Todd, Muslima S. Razaqyar, Jessica W. Witherspoon, Tokunbor A. Lawal, Ami Mankodi, Irene C. Chrismer, Carolyn Allen, Mary D. Meyer, Anna Kuo, Monique S. Shelton, Kim Amburgey, Dmitriy Niyazov, Pierre Fequiere, Carsten G. Bönnemann, James J. Dowling, Katherine G. Meilleur

**Affiliations:** ^1^Neuromuscular Symptoms Unit, National Institute of Nursing Research (NIH), Bethesda, MD, United States; ^2^Neurogenetics Branch, National Institute of Neurological Disorders and Stroke––NINDS (NIH), Bethesda, MD, United States; ^3^Division of Neurology, Hospital for Sick Children, University of Toronto, Toronto, ON, Canada; ^4^Department of Pediatrics, Ochsner Medical Center, New Orleans, LA, United States; ^5^Division of Neurology, Children’s of Alabama, Birmingham, AL, United States; ^6^Department of Paediatrics, Hospital for Sick Children, Toronto, ON, Canada; ^7^Department of Molecular Genetics, Hospital for Sick Children, Toronto, ON, Canada

**Keywords:** genotype, phenotype, *RYR1*, neuromuscular, magnetic resonance imaging

## Abstract

The ryanodine receptor 1-related congenital myopathies (*RYR1*-RM) comprise a spectrum of slow, rare neuromuscular diseases. Affected individuals present with a mild-to-severe symptomatology ranging from proximal muscle weakness, hypotonia and joint contractures to scoliosis, ophthalmoplegia, and respiratory involvement. Although there is currently no FDA-approved treatment for *RYR1-*RM, our group recently conducted the first clinical trial in this patient population (NCT02362425). This study aimed to characterize novel *RYR1* variants with regard to genetic, laboratory, muscle magnetic resonance imaging (MRI), and clinical findings. Genetic and histopathology reports were obtained from participant’s medical records. Alamut Visual Software was used to determine if participant’s variants had been previously reported and to assess predicted pathogenicity. Physical exams, pulmonary function tests, T1-weighted muscle MRI scans, and blood measures were completed during the abovementioned clinical trial. Six novel variants (two *de novo*, three dominant, and one recessive) were identified in individuals with *RYR1*-RM. Consistent with established *RYR1*-RM histopathology, cores were observed in all biopsies, except Case 6 who exhibited fiber-type disproportion. Muscle atrophy and impaired mobility with Trendelenburg gait were the most common clinical symptoms and were identified in all cases. Muscle MRI revealed substantial inter-individual variation in fatty infiltration corroborating the heterogeneity of the disease. Two individuals with dominant *RYR1* variants exhibited respiratory insufficiency: a clinical symptom more commonly associated with recessive *RYR1*-RM cases. This study demonstrates that a genetics-led approach is suitable for the diagnosis of suspected *RYR1*-RM which can be corroborated through histopathology, muscle MRI and clinical examination.

## Introduction

The ryanodine receptor isoform 1-related congenital myopathies (*RYR1*-RM) encompass a group of genetically and phenotypically diverse rare neuromuscular disorders that are estimated to affect approximately 1:90,000 pediatric individuals in the United States (1). Although *RYR1*-RM are considered slowly progressive, clinical presentation varies, they can include mild-to-severe symptoms ranging from delayed motor milestones, joint contractures, proximal muscle weakness, and fatigue to scoliosis, ophthalmoplegia, and respiratory insufficiency (2).

*RYR1* (19q 13.2) encodes a transmembrane calcium ion (Ca^2+^) channel (RyR1) which is embedded within the sarcoplasmic reticulum (SR) of skeletal muscle and plays an integral role in excitation–contraction coupling (3). *RYR1* is 160 kb in length and comprises 106 exons (4). As such, *RYR1* is one of the largest known human genes and has, in the past, posed a sequencing challenge. The homotetrameric RyR1 structure consists of four identical subunits ~565 kDa, which interact with other proteins and ligands, forming a macromolecular complex with an overall molecular mass of approximately 2.25 million Da (5). Causative *RYR1* variants are responsible for either chronic SR Ca^2+^ leak or impaired SR Ca^2+^ release. Both dominant and recessive modes of inheritance have been documented across the *RYR1*-RM spectrum, with the latter often associated with a more severe clinical phenotype (6, 7). *RYR1*-RM histopathological subtypes reported to date include central core disease (CCD; MIM# 117000), multi-mini core disease (MmD; MIM# 255320), centronuclear myopathy (CNM), congenital fiber-type disproportion (CFTD), and core-rod myopathy (CRM) (8–12). A range of *RYR1*-RM clinical phenotypes have also emerged more recently and include *RYR1* rhabdomyolysis-myalgia syndrome and atypical periodic paralysis (13, 14).

Central core disease is characterized by oxidative histological staining that reveals centralized absence of mitochondria and non-stained areas named “cores,” which are evident on muscle biopsy and can span the length of an entire muscle fiber (15). Genetically, CCD often occurs due to dominant or *de novo RYR1* variants although recessive cases have been reported (16, 17). Furthermore, CCD is considered allelic to malignant hyperthermia (MH) susceptibility (MIM# 145600), a potentially fatal pharmacogenetic condition which typically manifests following exposure of predisposed individuals to certain volatile anesthetics and muscle relaxants, including halothane and succinylcholine, respectively. Indeed, it is understood that 70–80% of MH cases may be directly related to “gain of function” *RYR1* variants. Although reported in 1:100,000 anesthetic procedures, this figure is likely an underestimate of true MH susceptibility as MH crises do not always occur upon initial exposure to a trigger (18, 19). Approximately 50% of those with *RYR1* variants are MH susceptible with such individuals often exhibiting central cores on biopsy (20).

Those affected by recessive *RYR1*-RM subtypes, such as MmD, are often clinically distinguishable from dominant cases by the presence of ophthalmoplegia and respiratory muscle weakness (10). Unlike CCD, in *RYR1*-related MmD, numerous amorphous cores of varying size are often evident on muscle biopsy and are the most commonly observed histopathological feature in recessive cases (10). MmD has also been associated with recessive variants in *SEPN1* which encodes the redox modulating glycoprotein selenoprotein N (21) as well as other myopathy-related genes, such as *MEGF10* and *TTN* (22, 23). In CNM, an abundance of centralized nuclei is the hallmark histopathological feature, with affected individuals exhibiting clinical features similar to MmD (24). In CFTD, NADH staining reveals hypotrophy of type-I (slow-twitch) fibers relative to type-II (fast-twitch) fibers (11). A CFTD histopathological diagnosis requires widespread observation of type-I fibers that are 35–40% smaller in diameter than type-II fibers (25). CRM is considered a severe congenital myopathy and is identified by the presence of cores as well as nemaline bodies on muscle biopsy that are otherwise characteristic of nemalin myopathy (MIM# 256030) (8, 26). Although variants in several other genes including *KBTBD13, ACTA1, NEB*, and *TMP2* have been linked to CRM, *RYR1* variants are considered the most common cause (27, 28). At present, there is not a clear ability to clinically distinguish patients with recessive variants by histopathological subtype alone.

The following study reports on six individuals with previously unreported *RYR1* variants that were identified as part of the first formal natural history study and clinical trial in this patient population. *RYR1*-RM histopathological subtypes discussed include CCD and CFTD.

## Materials and Methods

### Subjects

Novel *RYR1* variants were identified in six individuals with *RYR1*-RM (men *n* = 3, women *n* = 3; adult *n* = 2, pediatric *n* = 4) from five families that participated in the first formal natural history study and clinical trial in this patient population which tested the effects of *N*-acetylcysteine treatment on oxidative stress and fatigability (NCT02362425). All procedures associated with the trial were approved by the NIH Combined Neuroscience Institutional Review Board and all participants provided informed consent/assent prior to commencing the trial. Study participants included in this case report provided written informed consent for publication of their results. Eligibility criteria included (a) providing a genetic report confirming *RYR1*-RM, (b) being clinically symptomatic, and, if available, (c) presenting a muscle biopsy report indicative of *RYR1*-RM histopathology. Individuals attended baseline study visits at the National Institutes of Health (NIH) Clinical Center, Bethesda, MD, USA between March 2015 and June 2016.

### *RYR1* Sequencing and Variant Screening

For all six individuals, *RYR1* sequencing was conducted using whole blood at laboratories certified to the Clinical Laboratory Improvement Amendments (CLIA) prior to study enrollment. In Cases 1 and 2, Sanger sequencing of *RYR1* exons 1–106 was performed. In Case 3, Tier-1 partial Sanger sequencing of *RYR1* (exons 2, 6, 8, 9, 11, 11, 12, 14, 15, 17, 39, 40–41, 40–47, 95, and 100–104) was conducted. Exome sequencing was used to identify *RYR1* variants in Cases 4 and 6. In Case 5, targeted *RYR1* sequencing of exon 102 was performed, based on evidence of a familial variant in Case 3. Diagnostic genetic reports, detailing the abovementioned genetic testing, were obtained from participants medical records as part of the trial screening process. Variants causing nonsynonymous, nonsense, or splice-site substitutions and frameshift variants in the coding regions or splice signal sequences were screened using Alamut Visual software version 2.9.0 (Interactive Biosoftware, Rouen, France) by querying the nucleotide change provided by each laboratory. Alamut Visual complies with standards outlined by the American College of Medical Genetics regarding interpretation of sequence variants (29). Variants were considered novel if they were not previously reported in ExAC, ESP, HGVD, ClinVar, 1000 Genomes, or HGMD databases and not published in the scientific literature to date. Software tools (SIFT and PolyPhen-2) were also used to predict whether novel variants were likely to be deleterious.

### Physical Exam and Electrocardiogram

A full physical exam was performed by a Board-Certified Nurse Practitioner. The exam included anthropometric, head, eyes, ear, nose and throat, neurologic, respiratory, cardiovascular, abdominal, dermatological, psychiatric, and musculoskeletal assessments. A 12-lead electrocardiogram (ECG) was performed by a cardiologist for each individual.

### Blood Collection and Analyses

Phlebotomists obtained blood samples from the antecubital fossa of study participants following an overnight fast. Complete blood count (CBC) assessments were performed with whole blood using the bioanalyzer (Sysmex XN-3000, Sysmex America Inc., Lincolnshire, IL, USA). Creatine kinase, high-sensitivity C-reactive protein (hsCRP), and albumin concentrations were determined in plasma using a Cobas 6000 bioanalyzer (Roche, Branford, CT, USA).

### Pulmonary Function Tests

Pulmonary function tests (PFTs) were conducted in accordance with the American Thoracic Society Guidelines at the NIH Clinical Center Rehabilitation Medicine Department using a CPFS/D USB spirometer (MGC Diagnostics, Saint Paul, MN, USA) (30). Spirometry measures included forced vital capacity (FVC), forced expiratory volume at 1 s (FEV_1_), and maximal voluntary ventilation (MVV). BreezeSuite software was used to calculate % predicted values for the aforementioned measures (31). Respiratory insufficiency was defined as having <80% predicted FVC (32, 33). Those affected by neuromuscular disease may have difficulty performing the forcible exhalation required for the abovementioned PFTs (34). As such, slow vital capacity has been proposed as an alternative and was also measured in this study.

### Muscle Magnetic Resonance Imaging

A trained technician conducted muscle imaging at the NIH Radiology Department using a single 3-T whole-body magnetic resonance imaging (MRI) system (Verio, Siemens Medical Systems, Erlangen, Germany) and flexible phased array body-matrix coils. Axial images of the lower extremity muscles were acquired by T1-weighted (T1w) fast spin-echo and short tau inversion recovery (STIR) sequences with the following parameters: T1w, TR/TE = 600/20 ms, echo train length = 4, slice thickness/gap = 8 mm/20%, resolution = 1.4 × 1.0 mm, one average. STIR, TR/TE/TI = 5,600/82/220 ms, echo train length = 15, slice thickness/gap = 8 mm/20%, resolution = 1.2 × 1.0 mm, one average.

Acquired T1w images of the upper and lower legs were assessed for fatty infiltration in VUE Motion Software (Carestream Health Inc., Rochester, NY, USA) by a neurologist experienced in muscle MRI interpretation. The neurologist, blinded to participant information, assigned scores to images denoting whether each individual muscle was either affected, substantial fatty infiltration (score = 1); mild, evidence of intermediate fatty infiltration (score = 0.5) or spared, no fatty infiltration (score = 0). The most affected slice for the upper leg (thigh) and lower leg (calf) were used for scoring and interpretation.

### Histological Analyses

If available, muscle biopsy histopathology reports were obtained from participants’ medical records prior to study enrollment. Such reports were obtained for 4/6 cases. The typical workup included the following staining and histochemistry: NADH tetrazolium reductase (NADH-TR), hematoxylin and eosin (HE), Gömöri Trichrome (GO), periodic acid shift (PAS), Oil-Red O (ORO), cytochrome oxidase (COX), succinate dehydrogenase (SDH), ATPase, and myosin isoform (slow and fast heavy chain) immunohistochemistry.

## Results

**Figure 1:**
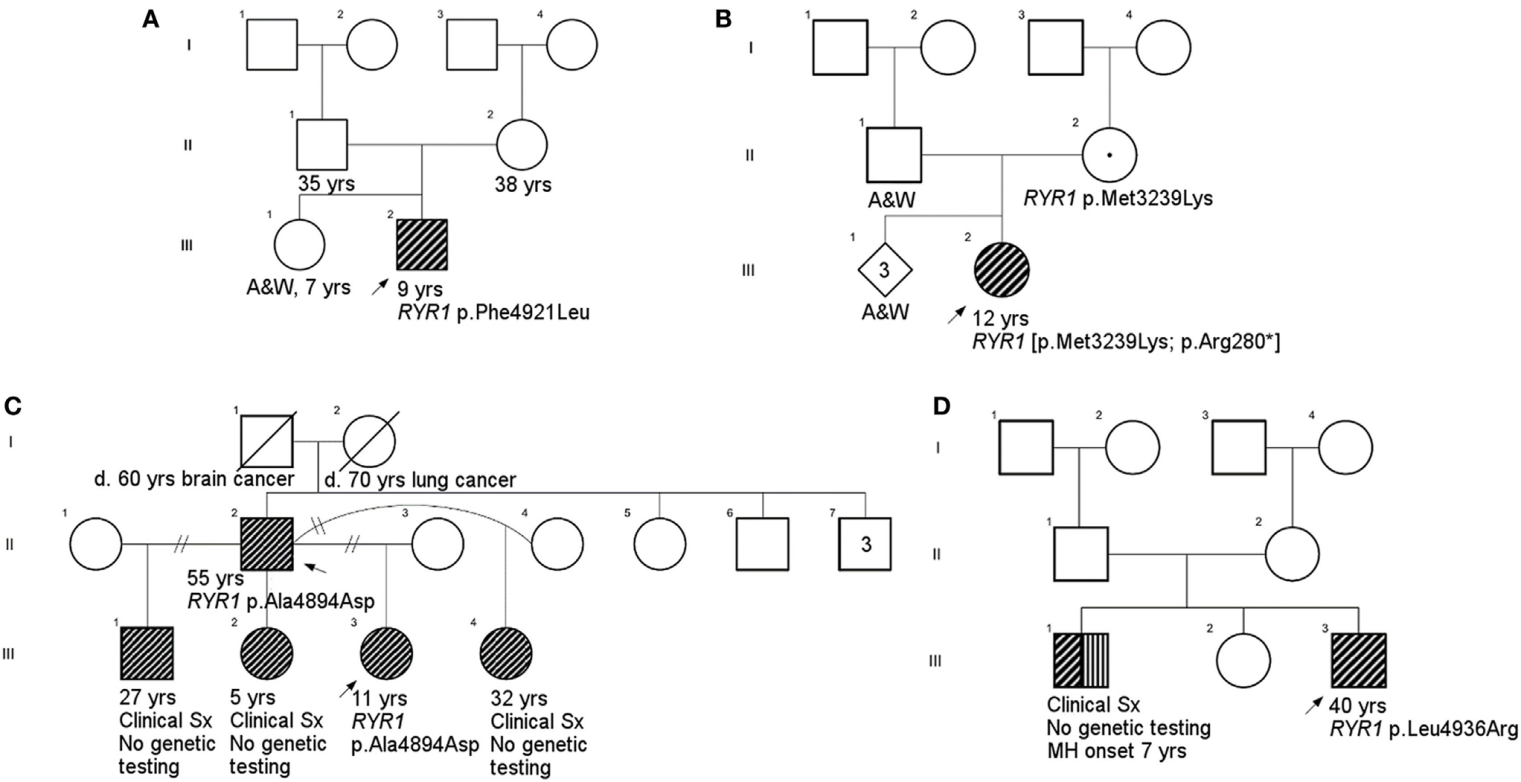
Family pedigrees. **(A)**, Case 1; **(B)**, Case 2; **(C)**, Cases 3 and 5 (father provided report of siblings other than proband, but they did not undergo physical examination as part of this study); **(D)**, Case 4. There was no family history available for Case 6 due to adoption. Squares and circles represent males and females, respectively. *RYR1*-RM-affected individuals are highlighted in diagonal shading. Individuals with a documented malignant hyperthermia are shown with vertical shading. Carriers are identified by a centralized period. Strike-throughs represent deceased family members and A&W refers to alive and well. Generations and birth order are identified by Roman numerals and Arabic numerals, respectively.

All individuals displayed clinical features consistent with *RYR1*-RM. An overview of the physical and biochemical characteristics of each case are provided in Table 1. Genetic, histological, and clinical features are presented in Table 2. Histopathological subtypes in this study included CCD (*n* = 2), CFTD (*n* = 1), and inconclusive CCD/MmD (*n* = 3). Pedigree charts for each family, detailing mode of inheritance, are provided in Figures 1A–D. Overall, muscle atrophy and impaired mobility with a Trendelenburg gait were the most common clinical symptoms and were identified in all cases (35). Bilateral proximal muscle weakness, particularly affecting the hip, was identified in all cases except Case 1. All cases were products of non-consanguineous relationships and were negative for a history of rhabdomyolysis.

**Table 1:** Physical and biochemical characteristics of individuals with novel *RYR1* variants.

	Case					
Measure	1	2	3	4	5	6
Sex	Male	Female	Female	Male	Male	Female
Age, year	7	10	9	39	54	7
Height, cm	114.7	150	127.7	183	170	138
Weight, kg	20.8	32.9	23.8	127	67.4	32.4
BMI, kg/m^2^	15.8	14.5	14.6	37.9	23.3	17
WBC, ×10^3^/μL	7.78	8.45	5.69	5.47	10.19	7.18
RBC, ×10^6^/μL	4.47	5.09	4.43	5.49	4.78	5.13
HGB, g/dL	12.3	11.5	12.5	15.6	16.3	14.7
HCT, %	36.9	37.2	38	44.7	48.1	43.4
MCV, fL	82.6	73.1	85.8	81.4	100.6	84.6
MCH, pg	27.5	22.6	28.2	28.4	34.1	28.7
MCHC, g/dL	33.3	30.9	32.9	34.9	33.9	33.9
MPV, fL	12.4	11	12.6	10.3	10.2	10.4
Albumin, g/dL	4.1	4.4	4.7	4.3	4.7	4.8
Creatine kinase, U/L	71	107	78	15	329	51
Total protein, g/dL	6.2	7.7	7.4	7.1	7.6	8.3
High-sensitivity CRP, mg/L	0.5	0.3	0.5	4.3	2.4	5.8

**Table 2:** Genetic, histological, and clinical characteristics of individuals with novel *RYR1* variants.

		Genetic characteristics				Muscle histology					Clinical features														

Case	Family	Exon or intron (E/I)	Nucleotide change	Amino-acid change	Mode of inheritance	Cores	Internal nuclei	Centralized nuclei	Type-I fiber predominance	Ambulatory	Abnormal gait pattern	Impaired mobility	Muscle weakness	Facial weakness	Ptosis	Muscle atrophy	Hypotonia	Contractures	Joint laxity	Scapular winging	Scoliosis	Impaired feeding	Respiratory impairment	Eye involvement	Rhabdomyolysis
1	1	E 102	c.14763C > G	p.Phe4921Leu	*De novo*	+	−	−	+	+	+	+	−	+	−	+	+	+	+	+	−	−	−	−	−
2^b^	2	E 10	c.838C > T	p.Arg280*	*De novo*	+	+	+	−	+	+	+	+	+	−	+	−	+	−	−	+	−	+	−	−
3	3	E 102	c.14681C > A	p.Ala4894Asp	Dominant	+	−	+	−	+	+	+	+	+	−	+	+	+	−	−	+	−	−	−	−
4	4	E 41	c.6697T > C	p.Cys2233Arg	Dominant	.	.	.	.	+	+	+	+	+	−	+	+	+	−	−	+	−	−	+	−
5	3	E 102	c.14681C > A	p.Ala4894Asp	Dominant	.	.	.	.	+	+	+	+	+	−	+	−	+	−	−	−	−	−	−	−
6	5	E 44	c.7166_7176del11	p.Asp2389Glyfs*16	Recessive^a^	−	+	−	+	+	+	+	+	+	+	+	+	+	−	+	−	+	+	+	−
		I 58	c.8933−1G > A	N/A (intronic)																					

*Period under muscle histology (.) indicates no biopsy report for notated case*.

*^a^Mode of inheritance based on clinical symptomatology*.

*^b^A previously reported variant was also identified in this case*.

### Case Histories and Current Clinical Features

In Case 1, decreased fetal movements were reported by the mother during pregnancy and gross motor delay was evident by 18 months of age. At age 3, this individual underwent an initial workup which included skeletal muscle biopsy, brain MRI, and bloodwork. Brain MRI and bloodwork, including CK, were normal; however, the biopsy revealed patchy loss of oxidative activity, and was interpreted as non-specific myopathy (inconclusive *RYR1*-RM histopathological subtype). Following this, lower extremity MRI revealed a pattern of muscle involvement indicative of *RYR1*-RM. Subsequent genetic testing identified the *RYR1* variant c.14763C > G (p.Phe4921Leu). Case 1 exhibited mild lower facial weakness and pectus excavatum that did not impair respiratory function. Musculoskeletal abnormalities were more pronounced with atrophic muscle mass affecting the quadriceps, mild scapular winging, and as generalized hypotonia. This was accompanied by ankle contractures and joint laxity at the elbows. Consistent with *RYR1*-RM clinical presentation, Case 1 completed the floor to stand maneuver with one-handed Gower’s and demonstrated Trendelenburg gait with an inability to run. No ECG abnormalities were observed and CBC, CK, hsCRP, and albumin values were within 1× the upper limit of normal (ULN). Absolute and % predicted PFT results were as follows: FVC 1.27 L, 92%; FEV_1_ 1.26 L, 101%; FEV_1_/FVC 0.99, 110%; SVC 1.21 L, 87%; and MVV 40 L, 84%.

Case 2 presented with difficulties running, climbing, and jumping at age 3, despite achieving the ability to walk at age 11 months. At 8 years of age, a muscle biopsy was performed and genetic testing identified the *RYR1* variants c.838C > T (p.Arg280*) and c.9716T > A (p.Met3239Lys). Case 2 exhibited bilateral, symmetric facial weakness and reported frequent headaches (one to two times per month) but was negative for epilepsy, stroke, cerebral palsy, hemiparesis, and dystonia. General hypotrophy was evident throughout, with the pectoralis particularly affected. Moreover, ankle contractures and scoliosis were observed, as well as one-handed Gower’s and Trendelenburg gait with impaired running ability. Bilateral, proximal, more than distal muscle weakness, was evident in her hips. Other than a prolonged QTcB interval (>440), ECG was normal and CBC, CK, hsCRP, and albumin values were within 1× the ULN. Absolute and % predicted PFT results were as follows: FVC 1.76 L, 78%; FEV_1_ 1.72 L, 87%; FEV_1_/FVC 0.97, 114%; SVC 1.94 L, 86%; and MVV 64 L, 68%.

At the time of study enrollment, the family history of Case 3 was pertinent for symptoms consistent with *RYR1*-RM; however, genetic testing had not been conducted on relatives. The father (Case 5) and two paternal half-brothers from different mothers were clinically symptomatic for *RYR1*-RM. The eldest half-brother, aged 25 years, required rod replacement for scoliosis and a skeletal muscle biopsy revealed multiminicores. First concerns regarding the physical function of Case 3 arose at 12 months of age. Initially, difficulty walking was attributed to a history of hip dyplasia; however, following no improvement with physical therapy, a skeletal muscle biopsy was conducted at 2 years of age. Negligible muscle tissue was obtained owing to fat replacement preventing a histopathological indication of *RYR1*-RM. Subsequent *RYR1* genetic testing revealed the variant c.14681C > A (p.Ala4894Asp). An additional skeletal muscle biopsy was obtained, at 4 years of age, on which core-like structures were identified. Case 3 had a history of fainting, in response to physical exertion, and asthma-related breathing difficulties but was negative for rhabdomyolysis. Bilateral, symmetric facial weakness was apparent with an inability to bury eyelashes. Musculoskeletal abnormalities included atrophic muscle mass affecting the quadriceps, generalized hypotonia, ankle contractures, and scoliosis. Bilateral, proximal muscle weakness was evident primarily in her hips, neck, and proximal upper and lower extremity muscles. A Trendelenburg, waddling gait was observed as well as one-handed Gowers’. A left-atrial abnormality was detected by ECG in Case 3 (sinus arrhythmia rate 61–94, V-rate variation >10% left-atrial abnormality, P,P′ > 60mS, <00.15 mV). CBC, CK, hsCRP, and albumin values were within 1× the ULN. Absolute and % predicted PFT results were as follows: FVC 1.65 L, 94%; FEV_1_ 1.29 L, 84%; FEV_1_/FVC 0.78, 89%; SVC 1.66, 94%; and MVV 57 L, 90%.

As a result of delayed gross motor milestones, such as the ability to hold up his head, Case 4 was initially diagnosed with Duchenne muscular dystrophy (DMD) at age 2 years. However, the clinical severity of Case 4 did not progress as quickly as expected given this diagnosis and therefore a skeletal muscle biopsy was obtained at 9 years of age. As per patient medical history, the biopsy results were inconclusive resulting in a broad rediagnosis of congenital myopathy. It was not possible for the participant’s healthcare provider to locate the biopsy report as the procedure was undertaken over 30 years ago. Suspected CCD prompted *RYR1* genetic testing which was conducted at 36 years of age. The c.6697T > C (p.Cys2233Arg) variant was identified. The family history was pertinent for a brother who had an MH crisis at 7 years of age resulting in brain hemorrhage. Case 4 exhibited strabismus and had a history of mild scoliosis. Bilateral, symmetric facial weakness was evident and accompanied by an inability to bury eyelashes. Muscle mass was atrophic though distribution evaluation was limited owing to subcutaneous fat tissue. Additional musculoskeletal findings included generalized hypotonia, and mild ankle contractures and weakness that bilaterally affect proximal and distal muscles. ECG was normal. CBC, CK, hsCRP, and albumin values were within 1× the ULN; however, CK concentrations were low (Table 1). Absolute and % predicted PFT results were as follows: FVC 3.37 L, 59%; FEV_1_ 2.96 L, 65%; FEV_1_/FVC 0.88, 110%; SVC 3.51, 61%; and MVV 121 L, 69%.

Case 5 (father of Case 3) became concerned regarding his physical function during childhood as he was unable to keep up with peers when running. As part of this study, targeted *RYR1* sequencing was conducted which resulted in identification of the *RYR1* variant c.14681C > A (p.Ala4894Asp). Mild facial weakness was evident and accompanied by a weak ability to bury eyelashes. Muscle mass was atrophic affecting the quadriceps; however, tone was normal. This individual had axial neck weakness, shoulder weakness, ankle contractures, difficulty heel walking, and an inability to run. A right-atrial abnormality was detected by ECG. CK was mildly elevated and CBC, hsCRP, and albumin were within 2× the ULN. Case 5 was also previously diagnosed with Wolff–Parkinson White at 38 years of age and had a recent history of recurrent melanoma over a period of 3 years. Absolute and % predicted PFT results were as follows: FVC 4.35 L, 98%; FEV_1_ 3.37 L, 98%; FEV_1_/FVC 0.77, 100%; SVC 4.41, 100%; and MVV 138 L, 100%.

Concerns regarding the physical function of Case 6 arose at 5 years of age when her mother (adoptive) noticed globally restricted eye movements. Case 6 had a history of severe hypotonia, finger contractures, and stiff elbows and knees since birth. While in the neonatal intensive care unit (NICU) for 4 months, a tracheostomy and feeding tube were placed owing to secretions and risk of aspiration. Due to ophthalmoplegia and suspected neuromuscular disease, a skeletal muscle biopsy and exome sequencing were undertaken at 1 year of age. The biopsy revealed fiber-type disproportion and exome sequencing identified *RYR1* variants c.7166_7176del11; (p.Asp2389Glyfs*16) and c.8933-1G > A. As well as ophthalmoplegia, Case 6 developed glaucoma as an infant. Speech therapy has been implemented to help with difficulty speaking and the tracheostomy and feeding tube remain in place. Facial weakness was bilateral, symmetric, and accompanied by tongue protrusion, a high-arched palate, and long facies. Quadriceps were atrophic with generalized hypotonia. Bilateral proximal, more than distal, skeletal muscle weakness primarily affected her hips and neck flexion. Ankle contractures, mild scapular winging, spinal rigidity, and a Trendelenburg gait were evident upon examination. Case 6 did not perform PFTs due to tracheostomy. No ECG abnormalities were observed and CBC, CK, and albumin values were within 2× the ULN. hsCRP concentration was elevated, likely owing to a concurrent ear infection (Table 1).

### Characterization of Novel Variants

**Figure 2:**
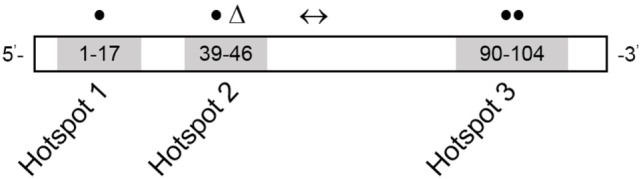
*RYR1* variant map showing the three hotspot regions. Numbers refer to exon range. Locations of variants reported in this study are depicted as follows: missense variants in closed circles, frameshift deletion in a triangle, and splice-site variant in a left–right arrow. Adapted from Ref. (15).

Novel variants are detailed in Table 2. Six novel variants were identified throughout *RYR1*, of which 5 were located in the traditional hotspot regions (15) (Figure 2). Based on recent cryo-EM reconstruction of the RyR1 structure (36, 37), two variants were in the transmembrane region, one was in the α-solenoid scaffold 1 (SPRY1) and three were in the bridging solenoid as follows; Case 1: The heterozygous variant, c.14763C > G (p.Phe4921Leu) in exon 102, located in the RyR1 transmembrane domain (36, 37), in this individual with CCD/MmD histopathology (Figure 1A). Case 2: The heterozygous variant c.838C > T (p.Arg280*) in exon 10, located in the RyR1 SPRY1 domain, was identified. This individual exhibited CCD/MmD histopathology and was also heterozygous for the previously reported *RYR1* variant c.9716T > A (p.Met3239Lys) in exon 66, located in the RyR1 bridging solenoid region (Figure 1B) (36, 37). Cases 3 and 5: The c.14681C > A (p.Ala4894Asp) *RYR1* sequence variant in exon 102 of *RYR1* was identified in a father (no biopsy) and daughter (CCD/MmD histopathology) (Figure 1C). This variant is located in the RyR1 transmembrane domain (36, 37). Case 4: The c.6697T > C (p.Cys2233Arg) variant in exon 41, located in the bridging solenoid region of RyR1 (36, 37), was identified in an individual with CCD histopathology and family history of MH (Figure 1D). Case 6: The 11 base pair deletion in exon 44 (c.7166_7176del11; p.Asp2389Glyfs*16), generating a premature stop codon in the bridging solenoid region of RyR1, and a second, intronic, splice-site variant (c.8933-1G > A) were identified in a child with CFTD histopathology (Case 6). Due to the absence of both family history and models expressing the abovementioned *RYR1* variants, thereby limiting the ability to conduct a complementation test, variants identified in Case 6 were deemed to be in translocation. This determination was therefore made based upon the individual’s clinical phenotype being consistent with previously reported for recessive *RYR1*-RM (11).

### Histopathology

Skeletal muscle biopsy reports were available for Cases 1, 2, 3, and 6. In Case 1, ATPase preparations (pH 4.3, 9.4) and myosin immunolabeling showed type-I fiber predominance. NADH, cytochrome c oxidase, and merosin staining was patchy in some fibers; however, other membrane labeling stains (spectrin, adhalin, dystrophin, dysferlin) were present. Fat clusters within fascicles replaced approximately 10–30% of fibers and this was confirmed by toluidine blue staining. Fibers at the periphery of the specimen exhibited large optically clear sarcoplasmic vacuoles and artifactual distortion. Electron microscopy did not reveal mitochondrial abnormalities or the presence of inclusion bodies. In Case 2, marked numbers of internal nuclei and type-I fiber predominance were observed on biopsy. Many nuclei were centralized, and fibers were both atrophic and hypertrophic with longitudinal disruption of cross striations. Oxidative stains revealed disruption of the myofibrillar architecture and cores were also apparent. On biopsy, Case 3 exhibited atrophic fibers of variable size and rare centralized nuclei. Oxidative staining revealed core-like structures; however, these were less well defined with ATPase staining. The biopsy for Case 6 revealed variability in fiber size and internalized nuclei. ATPase preparations (pH 4.3, 4.6, 9.4) showed that the largest fibers were type II (15–70 µM) and the smallest fibers type I (15–45 µM). There were no type-IIC fibers or fiber-type grouping. Type-I fibers comprised 74% of the fiber population.

### Muscle MRI

**Figure 3:**
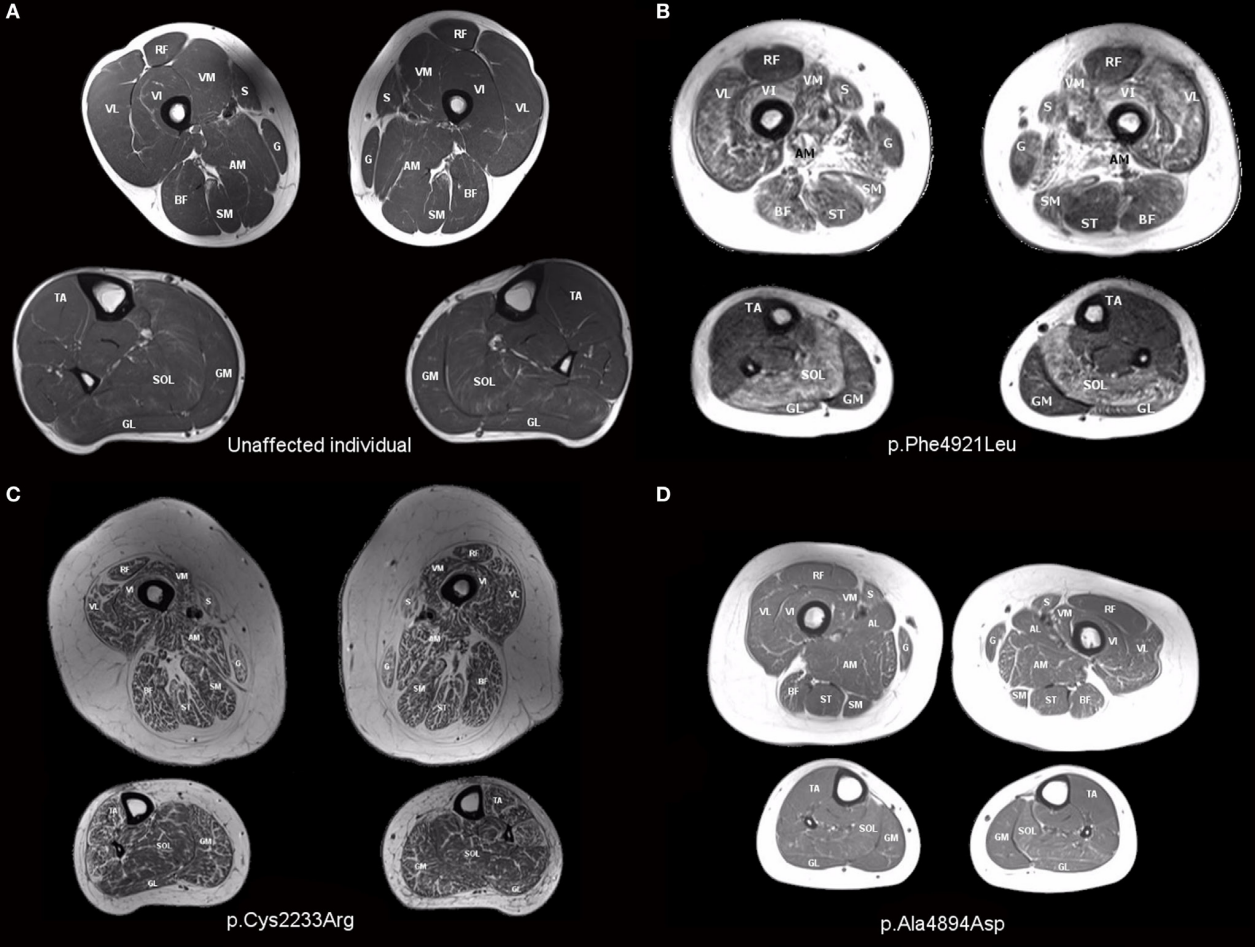
T1-weighted transverse MR images of the thigh and calf of an unaffected individual **(A)** and those with *RYR1*-RM **(B–D)**. **(B)** Case 1: selective muscle involvement typical of *RYR1*-RM with sparing of rectus femoris, gracilis, and tibialis anterior, **(C)** Case 4: global muscle involvement, and **(D)** Case 3: asymmetrical muscle involvement with the individual’s right side affected to a lesser extent than the left. This is particularly evident in biceps femoris and vastus lateralis.

Muscle involvement pattern was defined for each case per previously reported criteria for *RYR1*-RM (38, 39). Results for specific muscles are presented in Table 3, with fatty infiltration a prominent feature in all cases. Case 1 exhibited a typical *RYR1*-RM pattern of muscle involvement with relative sparing of rectus femoris (RF), adductor longus (AL), and gracilis (G), in the thigh, as well as tibialis anterior (TA) in the calf (Figure 3B). Cases 2 and 3 demonstrated muscle involvement that was consistent but did not strictly adhere to the typical pattern (Figure S1 in Supplementary Material; Figure 3D). In Case 3, selective involvement was observed in biceps femoris (BF) short head relative to long head. When comparing overall muscle involvement between the left and right sides, asymmetry was identified in Case 3 (left-side-affected more). Case 4 exhibited ubiquitous intramuscular fatty infiltration in both the thigh and calf (Figure 3C). As such, this case was not comparable to the aforementioned pattern; however, intramuscular fatty infiltration appeared to be greater, overall, in the thigh versus calf. Case 5 demonstrated a typical pattern of muscle involvement in the calf however; in the thigh, AL and G were deemed mild and affected, respectively, thus deviating from the typical pattern in the upper leg in that regard (Figure S2 in Supplementary Material). In Case 6 (Figure S3 in Supplementary Material), all muscles were affected by fatty infiltration; however, infiltration appeared to be to be less prominent in RF, G, and TA; muscles spared as per the typical *RYR1*-RM pattern.

**Table 3:** MRI pattern of fatty infiltration in the lower extremity muscles of individuals with RYR1-RM caused by novel variants^a^.

	Case					

Muscle	1	2	3	4	5	6
Vastus lateralis	Affected	Mild	Mild	Affected	Mild	Affected
Vastus medialis	Affected	Mild	Mild	Affected	Affected	Affected
Vastus intermedius	Affected	Mild	Mild	Affected	Mild	Affected
Rectus femoris	Spared	Spared	Spared	Affected	Spared	Affected
Sartorius	Mild	Affected	Affected	Affected	Affected	Affected
Adductor magnus	Affected	Mild	Mild	Affected	Affected	Affected
Adductor longus	Spared	Spared	Spared	Affected	Mild	Affected
Gracilis	Spared	Mild	Mild	Affected	Affected	Affected
Semitendinosis	Affected	Mild	Mild	Affected	Affected	Affected
Biceps femoris	Affected	Mild	Mild	Affected	Affected	Affected
Semi membranosis	Affected	Mild	Mild	Affected	Affected	Affected
Tibialis anterior	Spared	Spared	Spared	Affected	Spared	Affected
Gastrocnemius medialis	Mild	Mild	Mild	Affected	Affected	Affected
Gastrocnemius lateralis	Affected	Spared	Mild	Affected	Affected	Affected
Soleus	Affected	Mild	Mild	Affected	Affected	Affected

*^a^Fatty infiltration was scored as follows: spared (0), mild (0.5), or affected (1)*.

## Discussion

Over the past decade, advances in gene sequencing and a greater understanding of *RYR1*-RM clinical features have led to recognition that *RYR1*-RM manifest as vastly heterogenous neuromuscular diseases (40, 41). This study contributes to existing literature by characterizing genetic, laboratory, muscle MRI, and clinical findings of six symptomatic individuals with previously unreported *RYR1* variants.

The c.14763C > G (p.Phe4921Leu) variant identified in Case 1 is likely pathogenic as phenylalanine at this position is strictly conserved evolutionarily, and the substitution is predicted to affect the RyR1 protein structure and/or function (SIFT and PolyPhen-2). Two substitutions at this position, p.Phe4921Ser and p.Phe4921Thr, have both been previously associated with CCD (15, 42). Based on parental DNA testing, which was negative for variants in *RYR1*, the c.14763C > G (p.Phe4921Leu) variant identified in Case 1 was considered *de novo*. In Case 2, the *de novo* novel variant c.838C > T (p.Arg280*) was identified. A maternally inherited variant of uncertain significance, c.9716T > A (p.Met3239Lys), previously reported in the dbSNP database (rs371027185), was also identified in this individual. The father was negative for variants in *RYR1*. Both parents were otherwise healthy and there was no family history of myopathy. Although the p.Met3239 residue is moderately conserved among multiple species and substitution of an adjacent amino acid (p.Glu3238Gly) is reported to be causative for dominant, late-onset myopathy (43), there are currently insufficient allele frequency data to interpret its significance. Overall, the negative family history and truncating effect of c.838C > T (p.Arg280*) make it likely that this variant alone is causative (probably damaging, Polyphen-2); however, future research testing the functional effect of the p.Glu3238Gly variant is needed.

A father and daughter (Cases 5 and 3, respectively) both presented with the novel variant c.14681C > A (p.Ala4894Asp). Variants affecting the same amino acid have been previously reported as causative for congenital myopathy c.14680G > C (p.Ala4894Pro) (42), MH c.14680G > A (p.Ala4894Thr) (44), and CCD c.14681C > T (p.Ala4894Val) (44). This evidence strengthens the likelihood that the abovementioned variant is deleterious. Moreover, c.14681C > A (p.Ala4894Asp) is located in a domain that impacts ion transport and both SIFT and Polyphen-2 suggest that this variant is deleterious. In Case 4, the variant c.6697T > C (p.Cys2233Arg), located in MH/CCD hotspot region 2 (45), was identified. The location of this variant combined with the large physicochemical difference between Cys and Arg (Grantham distance: 180 [0–215]) make it likely that c.6697T > C (p.Cys2233Arg) is causative. SIFT and Polyphen-2 indicate that this variant is likely deleterious; however, in this individual’s case, genetic testing performed would not have captured any deletions, insertions, or duplications >18 base pairs in size so we cannot completely rule out a second variant. We mention this particularly because Case 4 had eye involvement, discussed in detail below. In Case 6, exome sequencing revealed the variant c.7166_7176del11 (p.Asp2389Glyfs*16) (probably damaging, Polyphen-2) and the splice-site variant c.8933-1G > A. Both are likely pathogenic due to truncation and disruption of a consensus splice site, respectively. Although functional studies were beyond the scope of the clinical trial from which data were obtained for this case series, the authors encourage future studies to include such analyses in order to elucidate conclusive implications of specific variants (46). This is particularly important since Schiemann and Stowell have demonstrated the limited sensitivity and specificity of predictive algorithms for *RYR1* variants, an issue also evident in the current case series (sensitivity 0.00–0.76; specificity 0.96–1.00).

Histopathological characteristics have long been used to differentiate between *RYR1*-RM subtypes, e.g., the presence of single or multiple cores on biopsy used to discern between CCD and MmD, respectively (47). Due to histopathological overlap, sampling challenges and variability between individual muscles (48), it is now well established that a diagnostic approach including genetics, muscle MRI, physical examination, and functional tests yields valuable information that can clarify inconclusive histopathological findings and *RYR1*-RM subtypes (49). Moreover, histopathological features are not necessarily static and have shown to vary, within individuals, over time (12, 50). Cases 1, 2, and 3 had inconclusive CCD/MmD biopsy reports with descriptive terms such as patchy reduction of cytochrome C oxidase activity, core-like structures, and disruption of myofibrillar architecture, making it difficult to distinguish between CCD and MmD based on this information alone. Nevertheless, reviewing biopsy findings in light of family history and physical examination make a CCD diagnosis plausible in these cases, owing to a milder clinical phenotype, MRI pattern, and absence of ophthalmoplegia and respiratory involvement (10). However, additional skeletal muscle biopsies would be required in order to reach this diagnosis conclusively.

Proximal muscle weakness and generalized hypotonia are considered hallmark features of *RYR1*-RM and have been reported consistently within the literature (51). Indeed, the current study identified these clinical features in the majority of cases, thereby supporting the established symptomatology. Although Case 5 exhibited an elevated CK concentration, hyperCKemia, defined as persistent CK elevations >1.5 times normal at >2 monthly intervals, was not absent from the case histories of individuals in this series (52). The muscle MRI data presented in this study corroborate the recognized *RYR1*-RM pattern of muscle involvement, in both dominant and recessive cases, but also draw attention to examples where this was either not evident or less apparent. In particular, there was no identifiable pattern in Case 4 owing to widespread fatty infiltration affecting all thigh and calf muscles. In Case 6, fatty infiltration affected all muscles although to a lesser extent in RF, G and TA. This demonstrates that complete sparing of specific muscles associated with the *RYR1*-RM pattern of involvement, such as in Case 1, may not always be apparent. Cases 4 and 6 also had the most severe clinical phenotypes, as evidenced by eye involvement. Case 4 exhibited strabismus, despite having only a single variant, and myopia. Strabismus was previously identified in a severe compound heterozygous case of CCD (16) and, as mentioned above, we cannot completely rule out the possibility of a second variant in this case. Case 6 presented with ophthalmoplegia. Ophthalmoplegia is considered an *RYR1*-RM clinical feature specific to recessive cases (28), a concept supported by the phenotype of Case 6. Two individuals with dominantly inherited *RYR1* variants exhibited respiratory insufficiency (FVC 78% and 59% predicted, Cases 2 and 4, respectively) in this study; a clinical feature typically associated with recessive cases at the severe end of the *RYR1*-RM clinical spectrum. Quinlivan and colleagues similarly reported a dominant case with cores on biopsy accompanied by respiratory insufficiency (32% predicted) and stated that this could therefore be considered an unusual case (53). Interestingly, in Case 2, SVC did not indicate respiratory insufficiency (86% predicted) whereas, in Case 4, respiratory insufficiency remained evident (61% predicted). Taken together, these findings suggest that respiratory insufficiency may more widespread across the *RYR1*-RM spectrum than previously recognized (54). The notable difference between % predicted FVC and SVC in Case 2 highlights the potential need to include SVC in the standard assessment of respiratory insufficiency in neuromuscular disorders, in order to rule out the confounding effect of spasticity (34). The use of SVC as an outcome measure in congenital myopathy clinical trials requires further research given its promise in other neuromuscular disorders (55). Case 6 was considered to have respiratory impairment, a phenotype consistent with the severe nature of *RYR1*-RM with CFTD histopathology, owing to difficulty managing heavy secretions which caused periodic airway restriction requiring frequent suctioning and eventual tracheostomy placement (56).

In conclusion, this study demonstrates that a genetics-led approach is suitable for the diagnosis of suspected *RYR1*-RM, which can be corroborated through histopathology, muscle MRI, and clinical examination. Next generation sequencing has made this approach possible for *RYR1* as well as other large genes where causative variants are responsible for neuromuscular disease (57). Given the complex genetic basis of *RYR1*-RM, and phenotypic overlap with other congenital myopathies, future studies may consider utilizing a congenital myopathy next-generation sequencing panel in order to rule out additional contributory variants in other genes. The genotype–phenotype data presented in this article will assist the diagnosis of specific *RYR1*-RM histopathological and clinical subtypes: a categorization which is essential for patient’s enrollment in clinical trials that target a specific myopathy.

## Ethics Statement

All procedures associated with the trial were approved by the NIH Combined Neuroscience Institutional Review Board and all participants provided informed consent/assent prior to commencing the trial.

## Author Contributions

JT collected data, interpreted results, and wrote the manuscript. KM, JW, CB, and JD conceived the study and contributed to data collection and interpretation. TL screened variants with JT and contributed to manuscript writing. AM and MM reviewed and graded MRI images. IC was the point of contact for patient recruitment and was involved in data collection. AK and MS recorded physical examination data. MS also contributed to manuscript writing. KA, DN, and PF made patient diagnoses and referred patients to the trial. All authors contributed to the interpretation of results and reviewed the final manuscript.

## Conflict of Interest Statement

The authors declare that the research was conducted in the absence of any commercial or financial relationships that could be construed as a potential conflict of interest.
