# Supplementary material for: Novel Variants in Individuals with RYR1-Related Congenital Myopathies: Genetic, Laboratory, and Clinical Findings
Source: Front Neurol. 2018 Mar 5;9:118. doi: 10.3389/fneur.2018.00118 (PMC5845096; doi:10.3389/fneur.2018.00118)
Supplement: Supplementary file 1 [file presentation_1.PDF]

Supplementary Material

Novel causative *RYR1* variants in six individuals with ryanodine receptor 1-related congenital myopathies: genetic, laboratory, muscle MRI and clinical findings

Joshua J Todd [*1], Muslima S Razaqyar [1], Jessica W Witherspoon [1], Tokunbor A Lawal [1], Ami Mankodi [2], Irene C Chrismer [1], Carolyn Allen [1], Mary D Meyer [1], Anna Kuo [1], Monique S Shelton [1], Kim Amburgey [3], Dmitri Niyazov [4], Pierre Fequiere [5], Carsten G Bönnemann [2], James J Dowling [3], Katherine G Meilleur [1].

*** Correspondence:** Joshua J Todd: joshua.todd@nih.gov

1 Supplementary Figures

Supplementary Figures

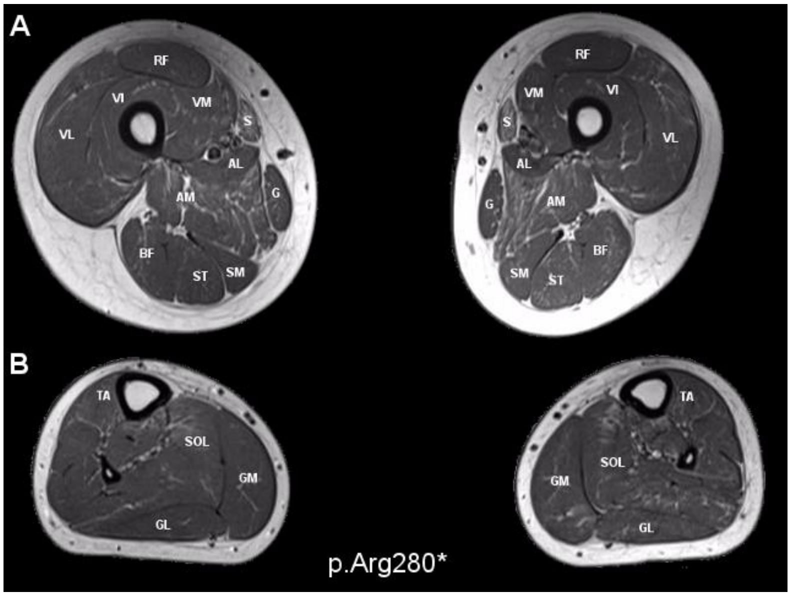

Figure S1: (Case 2). T1-weighted transverse MRI images of the thigh (A) and lower leg (B). In the thigh, S is affected. VL, VM, VI, AM, ST, BF, G and SM are mildly involved. Sparing is seen in RF and AL. In the lower leg, GM and SOL are mildly involved. TA and GL are spared.

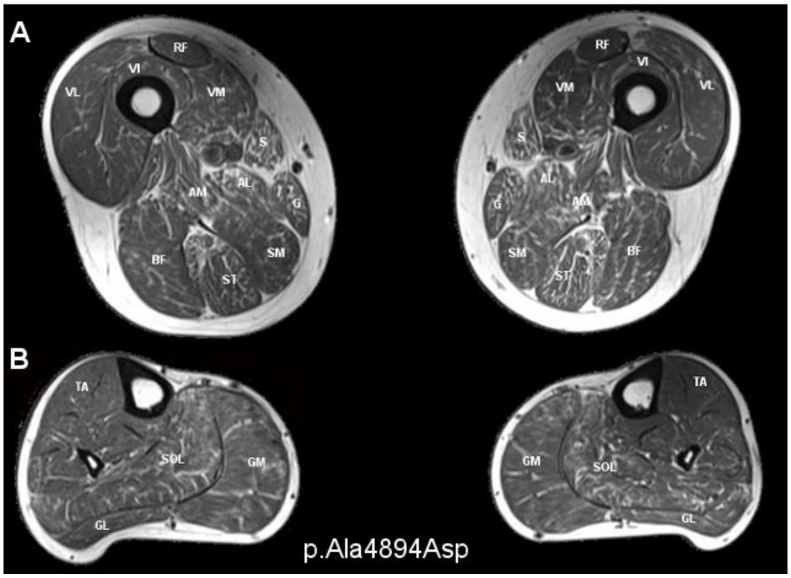

Figure S2: (Case 5). T1-weighted transverse MRI images of the thigh (A) and lower leg (B). In the thigh, VM, S, AM, G, ST, BF and SM are affected. VL and VI are mildly involved. Sparing is seen in RF. AL is not visible in the presented slice. In the lower leg, GM, GL and SOL are affected. TA is spared.

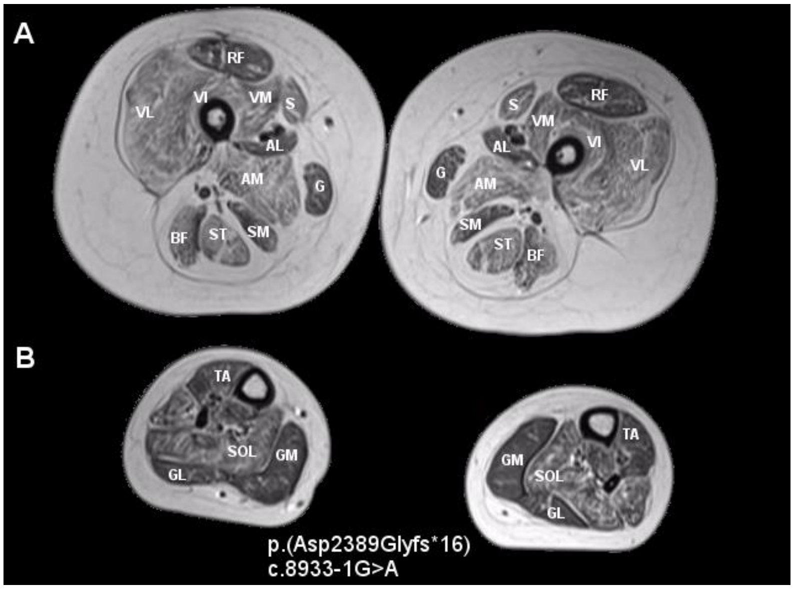

Figure S3: (Case 6). T1-weighted transverse MRI images of the thigh (A) and lower leg (B). In the thigh, RF, VM, S, AM, G, ST, BF and SM, AL, VL, and VI are affected. Fatty infiltration appeared to be to be less prominent in RF, G, and TA. In the lower leg, TA, GM, GL and SOL are affected.

Abbreviations: VL, Vastus Lateralis; VM, Vastus Medialis; VI, Vastus Intermedius; RF, Rectus Femoris; S, Sartorius; AM, Adductor Magnus; AL, Adductor Longus; G, Gracilis; ST, Semitendinosis; BF, Biceps Femoris; SM, Semi Membranosis; TA, Tibialis Anterior; GM, Gastrocnemius Medialis; GL, Gastrocnemius Lateralis; SOL, Soleus.
